# Detecting early onset of anthracyclines-induced cardiotoxicity using a novel panel of biomarkers in West-Virginian population with breast cancer

**DOI:** 10.1038/s41598-021-87209-8

**Published:** 2021-04-12

**Authors:** Hari Vishal Lakhani, Sneha S. Pillai, Mishghan Zehra, Benjamin Dao, Maria Tria Tirona, Ellen Thompson, Komal Sodhi

**Affiliations:** 1 Departments of Surgery and Biomedical Sciences, Marshall University Joan C. Edwards School of Medicine, Huntington, WV 25755 USA; 2 Division of Cardiology, Department of Internal Medicine, Marshall University Joan C. Edwards School of Medicine, Huntington, WV 25755 USA; 3 Department of Oncology, Edwards Comprehensive Cancer Center, Marshall University Joan C. Edwards School of Medicine, Huntington, WV 25755 USA

**Keywords:** Biomarkers, Oncology

## Abstract

Cardiotoxic manifestation associated with breast cancer treatment by anthracycline regimen increases patients’ susceptibility to myocardial injury, reduction in left ventricular ejection fraction and complications associated with heart failure. There is currently no standardized, minimally invasive, cost effective and clinically verified procedure to monitor cardiotoxicity post-anthracycline therapy initiation, and to detect early onset of irreversible cardiovascular complications. This study aims to create a panel of novel biomarkers and circulating miRNAs associated with cardiotoxicity, further assessing their correlation with cardiac injury specific markers, troponin I and T, and demonstrate the development of cardiac dysfunction in breast cancer patients. Blood obtained from West Virginian females clinically diagnosed with breast cancer and receiving anthracyclines showed upregulated level of biomarkers and circulating miRNAs after 3 and 6 months of chemotherapy initiation with increased levels of cardiac troponin I and T. These biomarkers and miRNAs significantly correlated with elevated troponins. Following 6 months of anthracycline-regimens, 23% of the patient population showed cardiotoxicity with reduced left ventricular ejection fraction. Our results support the clinical application of plasma biomarkers and circulating miRNAs to develop a panel for early diagnosis of chemotherapy related cardiac dysfunction which will enable early detection of disease progression and management of irreversible cardiac damage.

## Introduction

Chemotherapy related cardiac dysfunction (CRCD) is an incidental process that has been often associated with series of pathophysiological processes causing irreversible cardiac damage. While chemotherapeutic agents are mostly efficient in suppressing the cancerous growth, the underlying mechanisms operant compromises cardiac function and terminally differentiates cardiac outcome, due to excessive cardiotoxicities^1^. The untargeted chemotherapeutic intervention, with subsequent cardiotoxicities, increases patients’ susceptibility to myocardial injury, reduction in left ventricular ejection fraction (LVEF) and complications associated with heart failure^2^. The mitigation of CRCD and associated cardiotoxicity is particularly relevant in female patients afflicted with breast cancer. Breast cancer is globally prevalent, affecting over 2 million people, making it an overall third leading cause of cancer and a most common cause of cancer death in female population^3^. Due to the socio-economic disparities, the disease prevalence is unusually high specifically in West Virginia which is ranked 41st in the United States for the incidence of breast cancer^4^. According to West Virginia Department of Health & Human Resources (WV DHHR), approximately 25% of the West Virginian female population if affected with breast cancer.

Recent advances in the breast cancer treatment has led to improved outcomes for female patients with decreased overall mortality from this clinical condition. However, cardiotoxic manifestation associated with breast cancer treatment has resulted in increased cardiovascular complications post-chemotherapy, with cardiovascular diseases being the largest cause of death among breast cancer survivors^5^. The long-term cardiovascular morbidity and mortality associated with breast cancer treatment is attributable to complications including asymptomatic left ventricular (LV) dysfunction, congestive heart failure, pericarditis, myocardial ischemia, arterial hypertension, atrial and ventricular arrhythmias and thromboembolic disease^6^. CRCD in breast cancer can be acute, subacute, or chronic, with acute or subacute cardiotoxicity developing any time from the initiation of chemotherapy up to two weeks after the completion of the therapy^2^.

Cardiotoxicity is particularly relevant given the large number of female populations afflicted with breast cancer receive anthracycline therapy in the adjuvant setting^2^. The incidence of CRCD in the female population receiving anthracyclines alone has been reported to be about 4–36%, with 6% having clinically overt cardiotoxicity and 18% with subclinical cardiotoxicity^2^. Anthracyclines class of agents avert cellular division via modulation of DNA structure and ceasing its function^7^. Furthermore, these agents appear to affect cardiac function mainly through mechanisms that involve reactive oxygen species (ROS) formation, DNA damage through interaction with topoisomerase II and inhibition of protein synthesis^8^. Even though the cardiotoxicities associated with anthracycline agents have been widely implicated, the underlying cellular mechanism through which anthracyclines mediate this cardiotoxicity remains elusive.

It would be beneficial if the cardiotoxicity post-chemotherapy initiation could be monitored and attenuated prior to irreversible cardiovascular complications. The conventional approach and strategies proposed in the literature, to monitor anthracycline-induced cardiotoxicity, includes evaluation of LVEF at the beginning of the chemotherapy, with subsequent evaluation of cardiac function by echocardiography recommended post-chemotherapy initiation and completion^9–11^. However, there has been a limited implementation of these approaches in the standardized practice due to poor cost-effectiveness. Furthermore, these strategies are based on frequent and repeated echocardiographs for each cancer afflicted patient. However, the implications of echocardiography in the early prediction of the development of cardiac dysfunction due to cardiotoxicities is limited by its poor sensitivity and specificity, making it a less ideal screening tool for early intervention strategies^12–14^.

Based on a review of literature, we are proposing an alternate approach to the early diagnosis of CRCD by plasma biomarkers, which can detect cardiotoxicities before it is apparent on echocardiography and the presence of cardiovascular clinical symptoms. We propose a panel of biomarkers which have been implicated in myocardial injury, inflammatory response, and cardiac fibrosis and redox mechanisms associated with cardiovascular damage. These biomarkers include, myeloperoxidase (MPO), topoisomerase II beta (TOP2β), interleukin-6 (IL-6), matrix metalloproteinase -2 (MMP2) and -9 (MMP9). The role of micro RNAs (miRNAs), which are small single stranded RNA molecules, has been implicated in several diseases including cardiovascular diseases metabolic syndrome and diabetes^15^. These miRNAs are transported in the circulation and function in various pathways by altering gene expression through post-transcription modulation of messenger RNAs (mRNAs)^15^. Numerous studies have reported the use of miRNAs for pre-clinical diagnosis, since they are highly sensitive and specific in a diseased condition, while also exhibiting remarkable stability under harsh conditions^16,17^. Therefore, we further aim to assess circulating levels of several miRNAs including, miR-126, miR-34a, miR-499, miR-29a and miR-423, which has been demonstrated to play a critical role in stimulating cardiac remodeling, cardiac vasculature damage and contribute to the underlying pathological mechanisms involved in overall cardiac dysfunction. This study also aims to assess the levels of cardiac troponin I and T, which are specific markers for cardiac injury^18^ and correlate the changes in the troponins with the proposed biomarkers to demonstrate their specificity. This diagnostic modality using a panel of these biomarkers and miRNAs is preferable because of its minimally invasive method (a simple blood draw) and ease of use, cost effectiveness and most importantly, the timing of when these biomarkers are aberrant. Based on this biomarker panel, CRCD caused by cardiotoxicities may be detected earlier, which may allow for discontinuation of potentially cardiotoxic therapy and instituting heart failure medications earlier, which are both critical steps in addressing CRCD. This panel may potentially attenuate mortality rates in CRCD afflicted population, increase successful treatment outcomes and decrease overall healthcare costs.

## Results

### Demographics, echocardiography and clinical profile of anthracycline treated breast cancer patients

A total of 17 eligible female patients, with a new diagnosis of invasive ductal carcinoma, were enrolled for the study and completed the follow up protocol. Following their consent, all patients had blood withdrawn, laboratory panel assessed, and echocardiography performed at baseline (before initiation of anthracycline), 3- and 6-months after initiation of anthracyclines class of drug, doxorubicin. Another set of 17 healthy female subjects were also enrolled for the study to serve as controls. There was no significant difference in the mean age of the control and breast cancer patients. All patients diagnosed with breast cancer had triple negative status (ER−, PR− and HER2−) with an average tumor size of 2.8 cm (Table 1). The assessment of laboratory panel in these patients at baseline, 3- and 6-months showed no significant difference in the levels of albumin, alkaline phosphatase, alanine aminotransferase (ALT), aspartate aminotransferase (AST), total bilirubin, blood urea nitrogen (BUN), creatinine, total protein and prohormone B-type natriuretic peptide (proBNP) (Table 1). Similarly, echocardiographic assessment at baseline, 3- and 6-months also showed no significant difference in mean blood pressure, heart rate, LVEF, LV systolic and diastolic volume, LV stroke volume, cardiac output and cardiac index (Table 1). However, when the entire population was assessed independently, there were a total of four (23.5%) cardiotoxicity events at 6 months as defined by the Cardiac Review and Evaluation Committee. The LVEF among these patients declined by 8.1%, 7%, 9.3% and 11.5%, respectively. The reduction in LVEF in the patients who experienced cardiotoxicity events was noted at 6 months of anthracycline treatment from baseline.

**Table 1:** Summary of patient demographics, clinical profile and echocardiography parameters.

	Healthy controls	Invasive ductal carcinoma	
Sample size (n)	17	17	
Age (years)	61.2 ± 2.0	53.9 ± 3.0	
Tumor size (cm)	N/A	2.8 ± 0.4	

	Baseline	3 months	6 months
**Clinical data**			
Albumin (g/dL)	3.82 ± 0.07	3.65 ± 0.09	3.35 ± 0.2
Alkaline phosphatase (U/L)	78.8 ± 5.2	90.5 ± 9.1	77.6 ± 5.3
SGPT (ALT) (U/L)	27.0 ± 3.5	35.7 ± 4.5	28.1 ± 3.0
SGOT (AST)	19.5 ± 2.3	21.6 ± 2.8	20.7 ± 1.9
Bilirubin, total (mg/dL)	0.45 ± 0.06	0.58 ± 0.1	0.52 ± 0.1
BUN (mg/dL)	14.4 ± 1.5	12.3 ± 1.5	12.1 ± 1.2
Creatinine (mg/dL)	0.81 ± 0.05	0.80 ± 0.05	0.73 ± 0.04
Protein, total (g/dL)	7.5 ± 0.1	7.3 ± 0.2	6.6 ± 0.5
NT-proBNP (pg/mL)	94.5 ± 14.7	134.3 ± 19.2	136.3 ± 25.9
**Echocardiography**			
Systolic blood pressure (mmHg)	125.7 ± 2.8	127.9 ± 3.2	124.3 ± 2.9
Diastolic blood pressure (mmHg)	78.9 ± 1.7	77.9 ± 1.4	74.4 ± 1.9
Heart rate (bpm)	76.9 ± 3.1	82.7 ± 4.4	77.4 ± 3.7
LV ejection fraction (%)	62.9 ± 1.2	63.2 ± 0.9	61.5 ± 1.9
LV diastolic volume (mL)	76.1 ± 3.1	84.2 ± 3.8	77.1 ± 4.3
LV systolic volume (mL)	27.3 ± 1.0	30.6 ± 1.6	27.8 ± 2.2
LV stroke volume (mL)	48.8 ± 2.5	52.9 ± 2.7	49.3 ± 2.8
LV cardiac output (L/min)	3.8 ± 0.2	4.4 ± 0.4	3.8 ± 0.3
LV cardiac index (L/min/m^2^)	1.96 ± 0.12	2.36 ± 0.19	1.99 ± 0.12

The table summarizes basic characteristics of the study population with further assessment of key parameters from clinical profile and echocardiography in patients with invasive ductal carcinoma at each study interval of anthracycline treatment. There was no significant difference in the clinical and echocardiography parameters at any study interval, suggesting the prognostic efficacy of early progressive alterations in biomarker. Values represent means ± SEM.

### Assessment of plasma biomarker levels in anthracycline treated breast cancer patients

**Figure 1:**
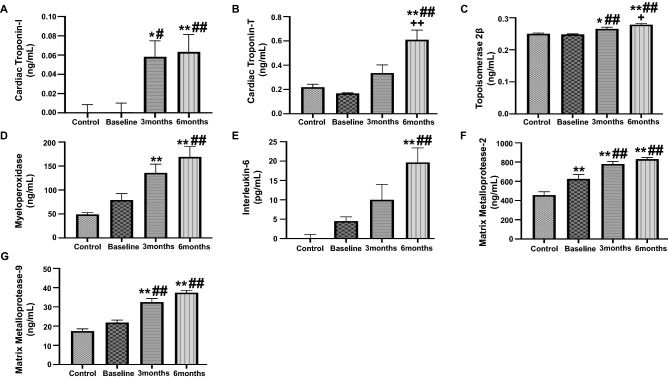
Quantitative analysis of plasma biomarkers in anthracycline-treated patient. Plasma concentrations of (**A**) Cardiac Troponin I, (**B**) cardiac Troponin T, (**C**) Topoisomerase 2β, (**D**) MPO, (**E**) IL-6, (**F**) MMP2 and (**G**) MMP9, assessed by ELISA. Values represent means ± SEM. *p < 0.05 vs. Control, **p < 0.01 vs. Control, ^#^p < 0.05 vs. Baseline, ^##^p < 0.01 vs. Baseline, ^+^p < 0.05 vs. 3 months, ^++^p < 0.01 vs. 3 months. Control (n = 17) and patients with breast cancer (n = 17) at baseline, 3 months and 6 months.

Blood was collected before initiation of anthracycline therapy, at baseline, and at 3- and 6-month follow up after anthracycline therapy initiation. The assessment of plasma biomarkers will provide an early insight into the slightest increase in cardiotoxicity in response to anthracycline treatment. We first evaluated the levels of high sensitive cardiac troponins, which are previously well-established markers of cardiac injury and strong independent predictors of cardiotoxicity. Our results showed significant elevated levels of cardiac troponin I at 3- and 6-months as compared to baseline and healthy controls (Fig. 1A). Furthermore, our results also showed a significant upregulation of high sensitive cardiac troponin T levels at 6 months after initiation of anthracyclines, as compared to control, baseline as well as 3 months (Fig. 1B). TOP2β have been previously implicated to play a role in anthracycline induced DNA damage, which contributes to cardiotoxicity. Our results showed a significant upregulation in the levels of TOP2β at 3 months, as compared to control and baseline (Fig. 1C). There was further increase in the levels of TOP2β at 6 months as compared to healthy control, baseline and 3 months (Fig. 1C). Oxidant stress and subsequent inflammation have been intricately linked to mechanism of anthracycline induced cardiotoxicity. To this end, our study showed significantly elevated levels of MPO, a marker of lipid peroxidation and oxidant stress, at 3 months as compared to control as well as at 6 months as compared to control and baseline (Fig. 1D). Subsequently, our results also showed significantly upregulated levels of IL-6, a marker of inflammation, at 6 months as compared to control and baseline (Fig. 1E). The level of key markers of cardiac remodeling and extracellular matrix damage, MMP2 and MMP9, were also significantly upregulated at 3 months and 6 months after the initiation of anthracycline treatment, as compared to control and baseline (Fig. 1F,G).

### Assessment of circulating miRNA expression in anthracycline treated breast cancer patients

**Figure 2:**
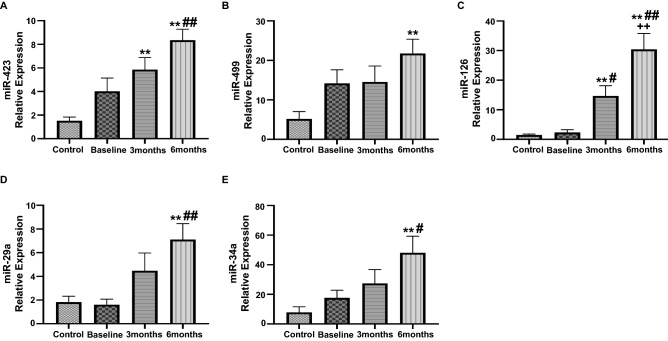
Assessment of circulating level of miRNAs in anthracycline-treated patient. Relative expression of circulating (**A**) miR-423, (**B**) miR-499, (**C**) miR-126, (**D**) mIR-29a and (**E**) mIR-34a, assessed by qRT-PCR analysis. Values represent means ± SEM. *p < 0.05 vs. Control, **p < 0.01 vs. Control, ^#^p < 0.05 vs. Baseline, ^##^p < 0.01 vs. Baseline, ^+^p < 0.05 vs. 3 months, ^++^p < 0.01 vs. 3 months. Control (n = 17) and patients with breast cancer (n = 17) at baseline, 3 months and 6 months.

The circulating miRNAs are critical in CRCD progression as they regulation the expression of genes associated with cardiac remodeling and myocardial injury, hence exacerbating the extent of cardiac damage. Our results showed significant upregulation in the expression of circulating miR-423 at 3 months as compared to control, which was further increased at 6 months, as compared to control and baseline (Fig. 2A). Furthermore, expression of miR-499 was also significantly upregulated at 6 months as compared to control (Fig. 2B). The relative expression of miR-126 was significantly upregulated at 3 months as compared to control and baseline (Fig. 2C). This increase in expression of miR-126 was further exacerbated at 6 months of anthracycline treatment as compared to control, baseline and 3 months (Fig. 2C). Next, we noted a significant upregulation in the relative expression of circulating miR-29a and miR-34a at 6 months after the initiation of anthracycline treatment in breast cancer patients (Fig. 2D,E). These results suggest that there was maximal upregulation in the expression of our panel of circulating miRNAs at 6 months after the start of study period offering the most prognostic efficacy at 6 months.

### Correlation analysis of plasma biomarkers and expression of circulating miRNAs with cardiac Troponin I and T

**Figure 3:**
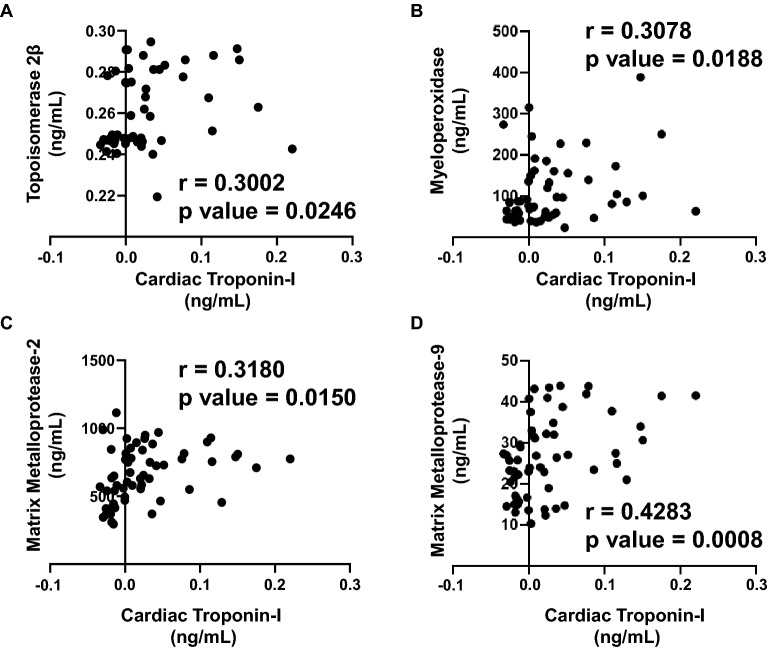
Correlation analysis of plasma biomarkers with high sensitive cardiac troponin I. Correlation was determined using Pearson’s *r* coefficient using two-tailed *p*-value to demonstrate significance (alpha = 0.05). Scatter dot plot between cardiac troponin I and plasma biomarkers showing statistical significance, which includes (**A**) Topoisomerase 2β, (**B**) MPO, (**C**) MMP2 and (**D**) MMP9. Each plot independently shows corresponding correlation coefficient (*r* value) and significance (*p* value).

**Figure 4:**
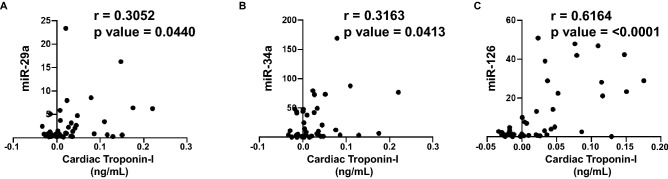
Correlation analysis of circulating miRNAs with high sensitive cardiac troponin I. Correlation was determined using Pearson’s *r* coefficient using two-tailed *p*-value to demonstrate significance (alpha = 0.05). Scatter dot plot between cardiac troponin I and circulating miRNAs showing statistical significance, which includes (**A**) miR-29a, (**B**) miR-34a and (**C**) miR-126. Each plot independently shows corresponding correlation coefficient (*r* value) and significance (*p* value).

**Figure 5:**
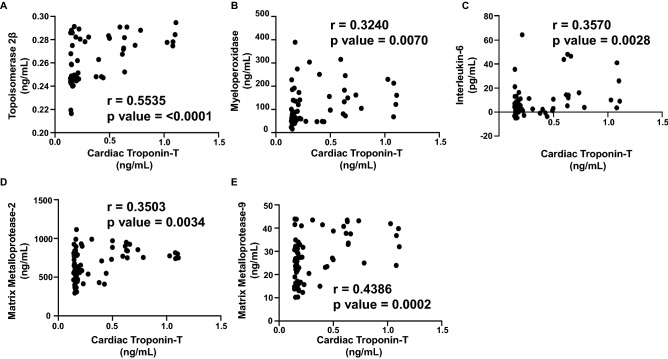
Correlation analysis of plasma biomarkers with high sensitive cardiac troponin T. Correlation was determined using Pearson’s *r* coefficient using two-tailed *p*-value to demonstrate significance (alpha = 0.05). Scatter dot plot between cardiac troponin T and plasma biomarkers showing statistical significance, which includes (**A**) Topoisomerase 2β, (**B**) MPO, (**C**) IL-6, (**D**) MMP2 and (**E**) MMP9. Each plot independently shows corresponding correlation coefficient (*r* value) and significance (*p* value).

**Figure 6:**
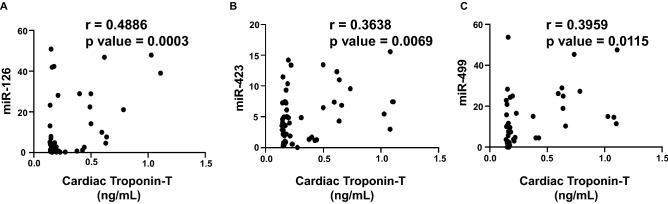
Correlation analysis of circulating miRNAs with high sensitive cardiac troponin T. Correlation was determined using Pearson’s *r* coefficient using two-tailed *p*-value to demonstrate significance (alpha = 0.05). Scatter dot plot between cardiac troponin T and circulating miRNAs showing statistical significance, which includes (**A**) mIR-126, (**B**) miR-423 and (**C**) miR-499. Each plot independently shows corresponding correlation coefficient (*r* value) and significance (*p* value).

Since cardiac troponin I and T are highly sensitive in response to the slightest changes in the LV function, as well as these markers are strong independent predictors of myocardial damage and cardiotoxicity, we aimed to establish a correlation with our panel of biomarkers and miRNAs. The extent of correlation was determined by the Pearson’s *r* coefficient. Each biomarker or circulating miRNA was independently plotted against cardiac troponin I and cardiac troponin T to determine which markers correlated the most during the anthracycline treatment up to 6 months. There was a significant positive correlation of cardiac troponin I with TOP2β (r = 0.3002) (Fig. 3A), MPO (r = 0.3078) (Fig. 3B), MMP2 (r = 0.3180) (Fig. 3C) and MMP9 (r = 0.4283) (Fig. 3D). The plasma levels of IL-6 did not show any significant correlation with cardiac troponin I (data not shown). Apart from that, there was also a strong positive correlation of cardiac troponin I with miR-29a (r = 0.3052) (Fig. 4A), miR-34a (r = 0.3163) (Fig. 4B) and miR-126 (r = 0.6164) (Fig. 4C). The circulating levels of miR-423 and miR-499 did not show any significant correlation with elevated cardiac troponin I (data not shown). Consequently, high sensitive cardiac troponin T was significantly correlated with TOP2β (r = 0.5535) (Fig. 5A), MPO (r = 0.3240) (Fig. 5B), IL-6 (r = 0.3570) (Fig. 5C), MMp2 (r = 0.3503) (Fig. 5D) and MMP9 (r = 0.4386) (Fig. (5E). In addition to that, high sensitive cardiac troponin T was also significantly correlated with miR-126 (r = 0.4886) (Fig. 6A), miR-423 (r = 0.3638) (Fig. 6B) and miR-499 (r = 0.3959) (Fig. 6C). The expression of circulating miR-29a and miR-34a apparently did not show any significant correlation with cardiac troponin T (data not shown).

## Discussion

Cardiotoxicity is the most common consequence in patients presenting with breast cancer receiving therapeutic anthracycline regimen, yet the pathophysiological cause remains elusive. Despite the presence of ample evidence supporting the notion of such cardiotoxicity, the lack of consensus guidelines to monitor cardiac function, at baseline and post-initiation, amplifies the risk of CRCD in patients undergoing chemotherapy. While the traditional approach in monitoring progressive decline in cardiac function by serial echocardiography detects significant changes in left ventricular function, it provides limited prognostic efficacy as well as limited sensitivity in monitoring early signs of myocardial injury, cardiac stress and the extent of cardiac remodeling^1^. Given these limitations as well as poor cost-effectiveness of the former approach, the present study aimed to identify a combination of novel plasma biomarkers and circulating miRNAs in a homogenous population of anthracyclines treated breast cancer patients, in order to demonstrate their joint utility in predicting cardiotoxicity. Patients enrolled in our study, receiving anthracyclines class of treatment, were monitored for progressive cardiac and systemic changes over the course of 6 months, starting at baseline (pre-initiation) with follow-ups at 3- and 6-months post-chemotherapy initiation. Our study reported significant reduction in LVEF at 6 months from baseline, in 23% of our patient population, the extent of which was similar to what has been previously reported in anthracyclines treated patients^19^. In addition, our study showed that 3- and 6-months following initiation of anthracyclines treatment, patients had elevated levels of cardiac troponin I and T, which are highly specific markers of myocardial lesions and cardiac injury^20^. These results were in concordance to previous findings as the cumulative line of evidence suggests that elevated levels of high sensitive cardiac troponin I and T are independent predictors of cardiotoxicity in patients receiving anthracycline therapy^21^. Since cardiac troponin I and T are specifically found in cardiac myocytes and a slightest myocardial injury can trigger the release of these troponins in the circulation, their upregulated levels may predict CRCD even before it is apparent on echocardiogram, hence suggesting their efficacy.

While there are several mechanisms operant in anthracycline induced cardiotoxicity, the excessive production of free radicals through mitochondrial redox cycling in cardiomyocytes and subsequent oxidant stress have been intricately linked in CRCD^22^. These changes have been associated with the upregulated levels of MPO, which is a pro-oxidant and pro-inflammatory marker, known to play a role in lipid peroxidation and acts as a strong predictor of CRCD in anthracycline treated patients^23,24^. Our results also showed upregulated levels of MPO at 3- and 6-months following initiation of anthracycline treatment, which was in concordance to previous studies which reported elevated levels of MPO starting at 3 months with an increasing trend even up to 15 months into anthracycline therapy^25^. This finding further corroborated our results that showed increasing levels of inflammatory cytokine, IL-6, following 6-months of anthracycline treatment from baseline. Previous studies have demonstrated that the upregulation of IL-6 with anthracycline treatment is associated with severe phenotypic changes in cardiomyocytes as well as significant decline in left ventricular function^26,27^, hence suggesting a strong efficacy of IL-6 as a prognostic biomarker. Furthermore, evidence suggests that the myocardial injury and phenotypic alterations in cardiomyocytes caused by oxidant stress are also mediated by upregulated levels of TOP2β^28^. Studies investigating anthracycline induced cardiotoxicity have suggested a significant role of TOP2β in causing double strand DNA-breaks leading to activation of p53 pathway eventually causing excessive ROS production^29^. Our findings were in concordance to these studies as our results showed upregulated levels of TOP2β at 3- and 6-months post-chemotherapy initiation from baseline, which may contribute to increased oxidant stress and eventual cardiac cell death due to cardiotoxicity. Apart from that, our study also demonstrated significant upregulation in the levels of plasma MMP-2 and MMP-9 biomarkers following initiation of anthracycline treatment. These findings were in concordance to previous studies that suggests the role of MMP-2 and -9 in cardiac remodeling as it aggravates ischemia, facilitates infiltration of neutrophils as well as causes degradation of extracellular matrix^12,30^. The effect of these biomarkers is mediated and further aggravated by the anthracycline induced oxidative stress and inflammation specifically in cardiomyocyte, resulting in compromised cardiac structure and left ventricular function decline^31^. Hence, the combination of these above biochemical plasma markers presents a strong prognostic utility due to their specific role in anthracycline induced cardiotoxicity.

Recent cumulative line of evidence suggests an important role of several circulating miRNAs in modulating cardiac function, hence an insight into their mechanistic action may allow for early risk assessment and their prognostic utility in determining cardiotoxicity. miR-34a is predominantly expressed in heart and their anthracycline induced upregulated expression has been associated with cellular apoptosis, cardiac regeneration as well as mediating oxidative stress, hence aggravating cardiotoxicity^32,33^. In concordance to previous reports, our results also showed significant upregulation of circulating miR-34a, following initiation of anthracycline treatment. Apart from that, our study also showed significant upregulated expression of miR-29a in anthracycline treated patients. The upregulation of plasma miR-29a has been previously reported to be associated with cardiac remodeling following cardiomyocyte injury as well as in patients with cardiac hypertrophy^32,34^. Apart from that, cardiomyocyte enriched miR-499 have been demonstrated to offer high sensitivity and specificity to myocardial injury and have been suggested to offer even superior early sensitivity than troponins^34,35^. The increased expression of miR-499 have been suggested as strong indicator of anthracycline induced acute cardiac injury^34^, which is in line with our findings that showed significant upregulated expression of miR-499 in anthracyclines treated patients. Similarly, miR-126 has been implicated as an early marker of heart failure with its upregulation mediated by anthracycline regimen could be potentially in response to associated cellular stress^36^. Our results also showed upregulation of plasma circulating miR-126 in response to anthracycline, which was in concordance to previous reports. Several studies have also reported an important role of upregulated miR-423 as an early prognostic marker for heart failure with overexpression of miR-423 associated with apoptosis in cardiomyocytes in response to anthracycline treatment^37,38^. Our study also showed upregulation of circulating miR-423 in patients receiving anthracyclines treatment, suggesting early molecular changes in cardiac tissues.

**Figure 7:**
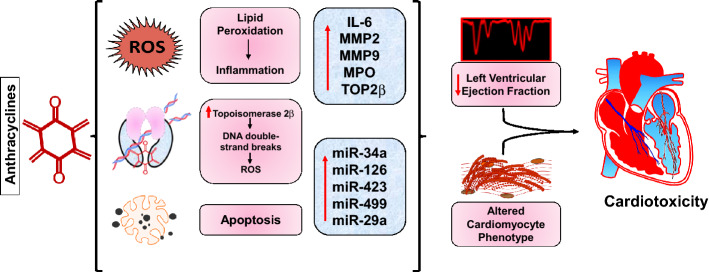
Schematic representation of anthracyclines induced cardiotoxicity. The treatment of breast cancer patients with an anthracycline regimen results in excessive ROS production with subsequent oxidant stress and inflammation in the cardiomyocyte. Anthracycline treatment also results in double strand DNA damage as well as cellular apoptosis which further contributes to the cardiotoxicity. The upregulation of biomarkers and circulating miRNAs associated with these pathological mechanisms operant in response to anthracycline treatment contributes to a decline in LVEF and altered cardiomyocytes, hence predicting early cardiotoxicity events.

In the present study, we further ascertained that the early changes in plasma biomarkers and circulating miRNAs were associated with subsequent cardiotoxicity by establishing a correlation of these markers with elevated levels of high sensitive cardiac troponin I and troponin T. Such approach using a statistical correlation is rather rational, since high sensitive cardiac troponin I and T are released specifically by cardiomyocyte and have been implicated by various studies as specific prognostic markers of cardiotoxicity with high sensitivity. Our results validated the prognostic utility of these biomarkers as TOP2β, MPO, IL-6, MMP-2 and -9 independently correlated with the elevated levels of cardiac troponin I and T. Furthermore, our results also demonstrated significant correlation of miR-29a, -34a, and -126 with cardiac troponin I, while miR-126, -423 and -499 correlated with cardiac troponin T. These findings provide further evidence that our proposed panel of biomarkers and miRNAs can effectively detect the early onset of anthracycline induced cardiotoxicity before it is even apparent on the echocardiogram, hence suggesting their clinical application in CRCD progression (Fig. 7).

While the findings presented in this study demonstrate a strong statistical significance and the translational applicability of the proposed biomarkers and circulating miRNAs in a clinical setting, there were several limitations of the study. The limitations of the study includes a small sample size and a shorter follow-up period (up to 6 months) for anthracyclines treatment in breast cancer patients enrolled for the study. Although our study showed that 23% population developed cardiotoxicity within 6 months of anthracycline therapy, the smaller sample size limited our ability to predict cardiotoxicity events in a larger population set. Despite the small sample size, the decline in LVEF and the percentage of cardiotoxicity events noted in our study was similar to previously reported findings that showed 21% of the population developed cardiotoxicity at 6 months of anthracycline treatment^19^, while another study noted cardiotoxicity in 24% of patients over a follow-up period of up to 15 months^25^. Apart from that, other potential limitations of the study include limited assessment of other sensitive indicators of left ventricular function such as longitudinal strain, lack of significance noted in other echo parameters that correlates with CRCD, which might be pertinent in bigger sample size and longer follow ups.

Together, the findings from the present study provides an important evidence for the efficacy of the proposed panel of biomarkers and circulating miRNAs in predicting the progressive decline in cardiac function in anthracyclines treated breast cancer patients. The combination of these biomarkers and their joint utility in a clinical setting provides a cost-effective and a non-invasive methodology in predicting cardiotoxic manifestation, hence improving morbidity and mortality. The utilization of this biomarker panel does not necessitate the termination of potential life saving cancer therapy. However, it does allow clinicians to monitor cardiac parameters in patients with highest risk, adjusting the dosage of the anthracycline regimen, as well as allow initiation of early intervention strategies to mitigate risks of cardiac function decline. While the proposed panel of biomarker will guide clinicians, an appropriate approach in mitigating the risk of cardiotoxicity events remains exclusive to the healthcare provider based on other major indices and confounding risk factors in each susceptible patient.

## Material and methods

### Study population

The Ethics Committee of the Cabell Huntington Hospital and Marshall University, West Virginia (IRB No.: 866164) approved the study. All research was performed in accordance with the guidelines and regulations set forth by the Declaration of Helsinki regarding the use and enrollment of human subjects in research. All patients and healthy subjects, serving as controls, gave written informed consent to participate in the study. A total of 17 Caucasian female patients, visiting the Edwards Comprehensive Cancer Center at Marshall University School of Medicine, were recruited for this study. All patients of age > 18 years or < 80 years having a new clinical diagnosis of invasive ductal carcinoma with triple negative status (estrogen receptor (ER−), progesterone receptor (PR−) and human epidermal growth factor receptor 2 (HER2−)), who were scheduled to receive only anthracyclines class of drug, doxorubicin, were included in the study. The anthracycline regimen, doxorubicin, was administered at 60 mg/m^2^ every 2 weeks for four cycles. Each patient was briefed about the use of the blood sample for this clinical study and each patient signed an informed consent. Patients wishing to enroll in the study were required to consent to give their blood sample and echocardiography at several intervals: prior to anthracyclines therapy (baseline; T0), 3 months (T1) and 6 months (T2) after the initiation of anthracyclines therapy. Patients with any second cancer (except for basal cell skin cancer and CIN), concurrent or prior chemotherapy and/or chest irradiation, history of myocardial infarction, cardiomyopathy, uncontrolled diabetes mellitus, uncontrolled hypertension, hereditary iron metabolism disorder, hyaluronan synthase 3 gene (HAS3) polymorphisms and age (> 80) were excluded from the study. Patients with LVEF of < 50%, as determined by echocardiography, as well as patients with symptomatic or asymptomatic heart failure were also excluded from the study. Patients taking any antihypertensive medications, antibiotics, weight loss medications or any other medication for a chronic disease were also excluded from the study. The patients’ follow up protocol was maintained with blood collection and echocardiography performed 3 months and 6 months after the initial diagnosis of invasive ductal carcinoma and the initiation of anthracycline chemotherapy for the assessment of biomarkers and cardiac function. All patients were assessed at 3 months and 6 months post-chemotherapy initiation for cardiotoxicity as defined by the Cardiac Review and Evaluation Committee with patients having: either a reduction in LVEF of ≥ 5% to < 55% from baseline with symptoms of heart failure or an asymptomatic reduction in LVEF of ≥ 10 to < 55% from baseline^19,25,39,40^.

Another set of patients was enrolled to account for healthy controls. A total of 17 healthy patients were also recruited, without clinical diagnosis of invasive ductal carcinoma or any form of cancer, cardiac dysfunction, diabetes, along with the exclusion criteria detailed above. Blood samples were collected once, and echocardiography was performed during their regular physician appointment. To ensure an appropriate selection of patients eligible for the study, trained hospital personnel examined patients’ medical records with appropriate confidentiality measures and in compliance with HIPAA.

### Blood samples

The patients enrolled for the study had venous blood withdrawn, as described previously^12,41,42^, at baseline (prior to anthracycline therapy), 3 months and 6 months after the initiation of anthracycline therapy, using standard protocol followed by trained hospital personnel. A total of approximately 10 mL of blood was withdrawn at each study interval, from the antecubital vein in the BD Vacutainer tubes for the analysis of biomarkers and measurement of circulating miRNA levels. Each blood sample obtained was processed within 30minutes of collection by centrifugation at 4000 rpm for 10 min under temperature setting of 4 °C. The aliquots were prepared for serum, separated from each blood sample and collected in appropriately labeled Eppendorf tubes, to avoid continuous freeze–thaw cycle. All aliquots were stored at − 80 °C which were further utilized for biomarker analysis and measurement of circulating miRNA levels.

### Quantification of biomarkers

Enzyme-Linked Immunosorbent Assays (ELISA) were performed for the quantification of serum biomarkers. The manufacturer’s protocol was followed for each of the following ELISA Kit: Human IL-6 ELISA (Abcam), Human MMP2 ELISA (Abcam), Human MMP9 ELISA (Abcam), Human Myeloperoxidase (MPO) ELISA (Abcam), Human Topoisomerase II beta ELISA (MyBioSource). ELISAs were also performed for human cardiac troponin I (Abcam) and human cardiac Troponin T (MyBioSource). These assays were performed in a specific manufacturer provided antigen coated 96-well plate and at the end of protocol, the plate was read at 450 nm wavelength, in BioTek ELx900 Absorbance Reader^12^. The standard curve was generated by plotting concentration of standards (x-axis) against corresponding absorbance (O.D value) of the standard (y-axis), as a XY-scatter plot to get 8-point curve. A linear trendline was added to the graph to achieve line of best fit with coefficient of determination (R^2^ value) of ≥ 0.98. The concentrations for each biomarker in each sample was calculated using the resulting equation from the line of best fit, as describe previously^12,41,42^.

### Expression of circulating miRNA levels

The manufacturer’s protocol was followed for total RNA extraction in serum samples, using miRNeasy Serum/Plasma Kit (Qiagen). Following RNA extraction, we used miRCURY LNA RT Kit (Qiagen) for our RT reactions, to prepare cDNA, with 50 ng of total RNA for each reaction. Further, miRNA specific primers were used, combined with SYBR green master mix, to perform RT-PCR reaction on 7500 HT Fast Real-Time PCR system (Applied Biosystems). Three technical replicates were used for each sample in the final qRT-PCR amplification data and their respective averages were used for relative fold change expression analysis. This protocol has been detailed previously^12,42^. The sequence of miRNAs is listed below:

- hsa-miR-34a-5p (UGGCAGUGUCUUAGCUGGUUGU);
- hsa-miR-29a-3p (UAGCACCAUCUGAAAUCGGUUA);
- hsa-miR-126 (UCGUACCGUGAGUAAUAAUGCG);
- hsa-miR-423-5p (UGAGGGGCAGAGAGCGAGACUUU);
- hsa-miR-499a-3p (AACAUCACAGCAAGUCUGUGCU).

### Transthoracic echocardiography

Transthoracic echocardiography was performed on healthy controls and patients at each study interval (baseline, 3 months and 6 months) with 2D Doppler and color flow imaging. Echocardiography was performed by a certified technician using Philip IE 33with a S transducer in an ICAEL-accredited laboratory^12^. All echocardiography measurements were obtained in accordance with guidelines set forth by the American Society of Echocardiography^43^. Echocardiography images were read by the physicians who were blinded to the rest of the study. Evaluation of ejection fraction (EF) was done by further 2D imaging which was calculated, as detailed previously^44^.

### Statistical analysis

The study was designed, conducted, recorded, analyzed and interpreted in an unbiased way, while ensuring that our results were reproducible. Data for each biomarker and expression of circulating miRNAs were analyzed using GraphPad Prism 7.0. Equal variance was assured by Bartlett’s test for each biomarker within each study interval. One-way ANOVA was performed to identify statistically significant differences in the mean serum levels for the different biomarkers. The Tukey post-hoc test was used to indicate which patient groups showed statistically significant differences for the biomarker levels measured. All data comparisons are presented at the non-significant (NS), p < 0.05 and p < 0.01 levels. Each bar represent values as means ± standard error of mean (SEM). To demonstrate a correlation between the cardiac injury specific markers, cardiac troponin I and T, a scatter plot was plotted for each biomarker and miRNA. A Pearson’s *r* coefficient determined the extent of correlation using a 95% confidence interval and choosing two-tailed *p-value* to determine significance (alpha = 0.05), as described previously^41^.
